# Comparing sputum microbiota characteristics between severe and critically ill influenza patients

**DOI:** 10.3389/fcimb.2023.1297946

**Published:** 2023-12-18

**Authors:** Zhixia Gu, Yuanyuan Zhang, Xue Zhao, Tingting Liu, Shugui Sheng, Rui Song, Ronghua Jin

**Affiliations:** ^1^ Beijing Key Laboratory of Emerging Infectious Diseases, Institute of Infectious Diseases, Beijing Ditan Hospital, Capital Medical University, Beijing, China; ^2^ Beijing Institute of Infectious Diseases, Beijing, China; ^3^ National Center for Infectious Diseases, Beijing Ditan Hospital, Capital Medical University, Beijing, China; ^4^ National Key Laboratory of Intelligent Tracking and Forecasting for Infectious Diseases, Beijing, China

**Keywords:** influenza virus, severe and critically ill, 16s-rDNA sequencing, bacteria, case control

## Abstract

**Background:**

Currently, limited attention has been directed toward utilizing clinical cohorts as a starting point to elucidate alterations in the lower respiratory tract (LRT) microbiota following influenza A virus (IAV) infection.

**Objectives:**

Our objective was to undertake a comparative analysis of the diversity and composition of sputum microbiota in individuals afflicted by severe and critically ill influenza patients.

**Methods:**

Sputum specimens were procured from patients diagnosed with IAV infection for the purpose of profiling the microbiota using 16S-rDNA sequencing. To ascertain taxonomic differences between the severe and critically ill influenza cohorts, we leveraged Linear Discriminant Analysis Effect Size (LEfSe). Additionally, Spearman correlation analysis was employed to illuminate associations between sputum microbiota and influenza Ct values alongside laboratory indicators.

**Results:**

Our study encompassed a total cohort of 64 patients, comprising 48 within the severe group and 16 within the critically ill group. Intriguingly, *Bacteroidetes* exhibited significant depletion in the critically ill cohort (*p*=0.031). The sputum microbiomes of the severe influenza group were hallmarked by an overrepresentation of *Neisseria, Porphyromonas, Actinobacillus, Alloprevotella, TM7x*, and *Clostridia_UCG-014*, yielding ROC-plot AUC values of 0.71, 0.68, 0.60, 0.70, 0.70, and 0.68, respectively. Notably, *Alloprevotella* exhibited an inverse correlation with influenza Ct values. Moreover, C-reactive protein (CRP) manifested a positive correlation with *Haemophilus* and *Porphyromonas*.

**Conclusion:**

The outcomes of this investigation lay the groundwork for future studies delving into the connection between the LRT microbiome and respiratory disorders. Further exploration is warranted to elucidate the intricate mechanisms underlying the interaction between IAV and *Alloprevotella*, particularly in disease progression.

## 1 Introduction

Influenza virus infection remains the primary cause of elevated incidence and mortality in respiratory infectious diseases globally (Nair et al., 2010; Iuliano et al., 2018; Li et al., 2019). Effective mitigation strategies, encompassing vaccines and antiviral drugs, play a pivotal role in alleviating the associated health and economic burdens (Cowling and Zhong, 2023; Kumari et al., 2023). However, challenges including vaccine-virus mismatches, suboptimal vaccine coverage, and the intricate interplay of influenza viruses with these countermeasures necessitate urgent exploration of innovative problem-solving approaches (Belongia et al., 2016; Nachbagauer and Palese, 2020; Morens et al., 2023).

In the past decade, modern technology has facilitated comprehensive investigation of host-associated microbiota (Cullen et al., 2020), revealing the pivotal role of microbiota composition in maintaining the equilibrium of healthy individuals (Dominguez-Bello et al., 2019). Conversely, dysregulation of microbial composition can exacerbate pathological conditions, attracting increased attention from research teams worldwide. In comparison with intestinal microbiota, our understanding of the post-influenza virus infection alterations in the respiratory tract microbial community is relatively limited. Nonetheless, the respiratory tract microbiota undeniably plays a pivotal role in shaping the host’s immune response and is crucial for effective eradication of invasive viruses.

Several studies have reported discernible differences in respiratory tract microbiota between healthy individuals and patients afflicted by respiratory virus infections (Korten et al., 2016; Kaul et al., 2020). The respiratory tract microbiota not only correlates with influenza symptoms and viral shedding, but also serves as a reliable predictor of influenza susceptibility (Lee et al., 2019a; Lee et al., 2019b; Tsang et al., 2020). Furthermore, investigations have linked the microbial composition in the respiratory tract to outcomes in influenza patients (Jia et al., 2017; Tsang et al., 2020) as well as other respiratory diseases (Haran et al., 2021; Rattanaburi et al., 2022). Simultaneously, research has demonstrated significant shifts in the microbial composition and diversity of the respiratory tract following influenza virus infection (Hanada et al., 2018; Wen et al., 2018; Zhou et al., 2020; Rattanaburi et al., 2022; Hernández-Terán et al., 2023). However, a majority of these investigations have primarily centered on the microbiota of the upper respiratory tract (URT), limiting insights from the lower respiratory tract (LRT) that could potentially enrich our comprehension of the role of microbiota in influenza-associated disease (Hernández-Terán et al., 2023).

In light of these considerations, this study endeavors to employ 16S rRNA gene sequencing to meticulously compare the diversity and configuration of LRT microbiota in severe and critically ill influenza patients. Additionally, we seek to scrutinize the correlation between respiratory tract microbiota and the Ct value, alongside laboratory examinations, thereby attaining a deeper insight into the intricate interplay of respiratory tract microbiota in influenza patients.

## 2 Materials and methods

### 2.1 Study subjects

A total of 64 individuals with confirmed influenza were enrolled from December 2018 to January 2020 at Beijing Ditan Hospital, Capital Medical University. Among them, 48 individuals exhibited severe influenza symptoms, while 16 were classified as critically ill influenza patients. Severe in’

M-luenza cases met one or more of the following criteria: (1) persistent fever exceeding 3 days, coupled with intense cough, purulent or hemoptysis sputum, or chest pain; (2) rapid and labored respiration, dyspnea, and cyanosis of the lips; (3) altered cognitive function, including delayed responsiveness, drowsiness, restlessness, or seizures; (4) severe emesis, diarrhea, and signs of dehydration; (5) concurrent pneumonia; (6) considerable aggravation of pre-existing chronic ailments; (7) other clinical conditions necessitating hospitalization. Critically ill influenza patients met one or more of the following conditions: (1) progressive respiratory insufficiency requiring mechanical ventilation; (2) shock; (3) acute necrotizing encephalopathy; (4) multi-organ dysfunction; (5) other grave clinical situations mandating close monitoring and treatment. Exclusion criteria encompassed individuals below 18 years of age and pregnant women.

### 2.2 Sputum samples collection and DNA extraction

Prior to sample collection, participants were instructed to rinse their mouths, and dentures or braces were to be removed. Sputum was collected following a deep cough. Samples were deemed unacceptable if saliva and food residues accounted for over two-thirds of the sputum volume or if saliva and oral contaminants constituted more than half of the sputum volume. Each sputum sample, obtained from participants upon admission or in the early hours of the second day of admission, amounted to 2 ml. Samples were preserved in sterile containers at -80°C until DNA extraction, which was performed using the MagaBio Pathogens DNA/RNA Purification Kit (BSC75S1E) following the manufacturer’s guidelines. The concentration, purity, integrity, and size of DNA were determined through NanoDrop (Thermo Fisher Scientific) and 1.0% agarose gel electrophoresis. Subsequently, the DNA samples were frozen at -20°C for subsequent analyses.

### 2.3 Identification of influenza A/B and 16S ribosomal RNA Gene sequencing

Influenza A virus (IAV) identification was executed for all sputum samples using the Influenza A/B virus nucleic acid detection kit (PCR fluorescent probe method, Applied Biological Technologies, Beijing, China). The bacterial 16S rRNA gene sequences encompassing the variable regions V3–V4 were amplified employing the primers 341F (5’-CCTACGGGNGGCWGCAG-3’) and 805R (5’-GACTACHVGGGTATCTAATCC-3’), alongside the Q5 High-Fidelity 2X Master Mix (New England BioLabs Inc., Ipswich, MA). The resulting products underwent purification using 0.9× volumes of AMPure XP beads (Beckman Coulter, Inc., Brea, CA). Each sample was quantified using the Qubit 2.0 Fluorometer (ThermoFisher Scientific, Inc., Waltham, MA), pooled with equal input mass, and subjected to further purification using 0.9× volumes of AMPure XP beads (Beckman Coulter, Inc., Brea, CA). The final sequencing pool’s concentration was determined by qPCR using the KAPA Library Quantification Kit (KAPA Biosystems, Wilmington, MA) on a Roche 480 LightCycler (Roche, Basel, Switzerland). Subsequent sequencing was conducted by Beijing Novogene Technology Co. employing an Illumina PE 250 on an Illumina Noveseq Sequencer (Illumina, Inc., San Diego, CA).

### 2.4 Bioinformatics and statistical analyses

High-throughput 16S rRNA sequencing raw fastq files were demultiplexed and subjected to quality filtering using QIIME (version 2022.8.0). Dada2 was employed to truncate the linker sequence, merge the paired ends, eliminate chimeras, and generate amplicon variant sequences (ASVs) for noise reduction. Taxonomic analysis of each 16S rRNA gene sequence was performed using QIIME (version 2022.8.0) and compared against the SILVA rRNA database with a confidence threshold of 70%.

Descriptive statistics were utilized to represent continuous variables as mean (standard deviation [SD]) and categorical variables as frequency (percentage). A comparison of patient characteristics between the severe and critically ill influenza groups was achieved using the t-test or the Wilcoxon rank-sum test (continuous variables), and the Chi-square test or Fisher’s exact test (categorical variables). Relative abundance was calculated as the proportion of a specific bacterium relative to the total bacterial count in a given sample. Statistical analyses were carried out using R 4.3.1. A p-value less than 0.05 denoted statistical significance. The Wilcoxon rank-sum test was employed to assess differences in alpha diversity indices between the two groups. Beta diversity was evaluated via principal coordinate analysis (PCoA) to ascertain sample group similarities. Nonparametric multivariate analysis of variance (Adonis) was utilized to test inter-group differences. Linear discriminant analysis effect size (LEfSe) was conducted to identify bacteria accounting for distinctions between the two sample groups, using a logarithmic LDA score threshold of 2.0.

## 3 Results

### 3.1 General clinical features of the patients

The study encompassed 48 individuals diagnosed with severe influenza and 16 individuals categorized as critically ill influenza patients in a cross-sectional analysis. None had received influenza vaccination. Essential demographic attributes such as gender, age, smoking habits, and alcohol consumption were effectively matched between the two groups. No discernible differences emerged in underlying conditions (hypertension, diabetes, hyperlipidemia, cerebral vascular disease, heart disease, and chronic pulmonary disease) between the two groups (*p*=1.000, 0.106, 1.000, 0.427, 0.282, 0.521, respectively). Only one of the 64 patients was first diagnosed in our hospital, and most of the patients had antiviral treatment history before the treatment in our hospital, but there was no significant difference in the history of antiviral treatment and the days from onset to admission between the two groups (*p*=1.000, 0.764, respectively) (**Table 1**).

**Table 1:** General clinical characteristics of influenza patients.

Characteristics	Severe (n=48)	Critically ill (n=16)	statistic	*p*-value
Age (years)	65.38 ± 2.43	70.38 ± 2.69	t=-1.379	0.175
Gender (male/female)	24/24	9/7	χ^2 = ^0.188	0.665^&^
Smoking, N (%)	8 (16.67)	2 (12.50)	χ^2 = ^0.000	1.000^#^
Drinking alcohol, N (%)	8 (16.67)	1 (6.25)	χ^2 = ^0.388	0.533^#^
Hypertension	21 (43.75)	7 (43.75)	χ^2 = ^0.000	1.000^&^
Diabetes	7 (14.58)	6 (37.50)	χ^2 = ^2.606	0.106^#^
Hyperlipidemia	7 (14.58)	2 (12.50)	χ^2 = ^0.000	1.000^#^
CVD	6 (12.50)	4 (25.00)	χ^2 = ^0.632	0.427^#^
Heart disease	14 (29.20)	7 (43.80)	χ^2 = ^1.158	0.282^&^
Chronic pulmonary disease	12 (25.00)	6 (37.50)	χ^2 = ^0.412	0.521^#^
External hospital visits before this visit	47 (97.9)	16 (100)	χ^2 = ^0.000	1.000^&^
Antiviral treatment in external hospitals	41 (85.4)	14 (87.5)	χ^2 = ^0.000	1.000^&^
WBC (4-10*10^9^/L)	6.35 (4.10, 8.86)	8.88 (6.74, 10.81)	Z=-2.295	0.022
NE% (50-70%)	73.94 (64.27, 81.21)	86.36 (76.31, 89.74)	Z=-2.837	0.005
NE# (2-8*10^9^/L)	4.74 (2.38, 7.08)	7.32 (5.42, 8.45)	Z=-2.388	0.017
LY% (20-40%)	18.22 (11.59, 24.17)	9.67 (6.64, 13.33)	Z=-2.706	0.007
LY# (1-5*10^9^/L)	1.14 (0.73, 1.52)	0.78 (0.56, 1.05)	Z=-2.000	0.045
MO% (3-8%)	7.50 (5.20, 11.18)	4.12 (2.07, 8.37)	Z=-2.628	0.009
BA% (0-1%)	0.20 (0.05, 0.32)	0.00 (0.00, 0.10)	Z=-2.854	0.004
BA# (0-0.1*10^9^/L)	0.01 (0.00, 0.02)	0.00 (0.00, 0.01)	Z=-2.407	0.016
CRP (0-6*10^9^/L)	54.35 (31.22, 149.85)	117.65 (41.97, 216.60)	Z=-1.147	0.251
Ct value^$^	23.80 (20.40, 29.63)	21.64 (17.98, 25.92)	Z=-1.628	0.104
Hospitalization days of this visit	7.00 (5.00, 9.75)	8.00 (6.00, 10.75)	Z=-1.647	0.100
Days from onset to admission	3.00 (2.00, 5.00)	3.00 (2.00, 5.75)	Z=-0.300	0.764
Days from onset to sample collection	4.00 (2.00, 5.75)	3.00 (2.25, 5.75)	Z=-0.542	0.588

^#^: Continuity correction χ^2^ test.

^&^: Pearson’s chi-squared test.

^$^: Ct value less than or equal to 36 is considered positive.

CVD, Cerebral Vascular Disease; WBC, White Blood Cell; NE%, Neutrophil percentage; NE#, Neutrophil count; LY%, Lymphocyte percentage; LY#, Lymphocyte count; MO%, Monocytes percentage; BA%, Basophil percentage; BA#, Basophil count; CRP, C-reactive protein.

Each sputum and blood sample obtained from participants upon admission or in the early hours of the second day of admission. There were no statistical differences about the duration between the onset of illness and sample collection (*p*=0.588). Within the critically ill group, elevated levels of white blood cells (WBC), neutrophil percentage (NE%), and neutrophil count (NE#) were observed in comparison to the severe group (*p*=0.022, 0.005, 0.017, respectively). Conversely, lymphocyte percentage (LY%), lymphocyte count (LY#), monocyte percentage (MO%), basophil percentage (BA%), and basophil count (BA#) exhibited lower values in the critically ill group as opposed to the severe group (*p*=0.007, 0.045, 0.009, 0.004, 0.016, respectively) (**Supplementary Figure 2**). Although no statistically significant disparities emerged in C-reactive protein (CRP) and Ct values between the two groups (*p*=0.251, 0.104, respectively), the critically ill group exhibited heightened CRP levels compared to the severe group, while the Ct value in the critically ill group was lower than that in the severe group. In addition, in this diagnosis and treatment, there was no statistically significant difference in the length of hospital stay between the two groups (*p*=0.100), with two patients in the critically ill group dying on days 6 and 19 after admission (**Table 1**).

We conducted a careful review of each patient’s medical record system, collating all respiratory pathogens tested during hospitalization except influenza virus. This included: (1) Sputum bacterial, fungal and *Haemophilus* cultures: *Pseudomonas aeruginosa* and *Haemophilus influenzae* were detected in samples from two critically ill patients respectively, and *Klebsiella pneumoniae subsp. Pneumoniae* was detected in the sample from a severe patient. (2) Sputum acid-fast staining: All the samples were negative or did not undergo this examination. (3) *Mycoplasma pneumoniae* antibody (gelatin particle agglutination assay) and *Mycoplasma pneumoniae* nucleic acid test: A total of 37 patient samples were tested for the former (12 showed positive antibodies), but the nucleic acid tests were negative, which may mean that the patients had been infected with *Mycoplasma pneumoniae* but are not currently infected. (4) Blood culture: All samples were negative or not performed.

### 3.2 Analysis of sputum microbial diversity in the severe and critically ill influenza groups

**Figure 1:**
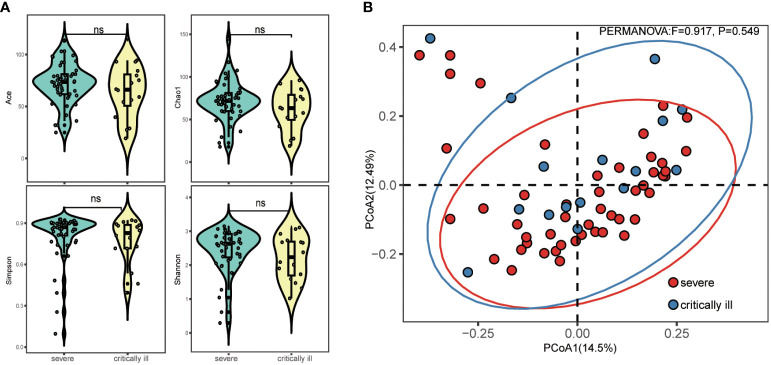
Alpha and beta diversity of sputum microbiota in the severe and critically ill influenza patients. **(A)** Alpha diversity (Simpson’s index of diversity, Shannon index, Chao1 and ACE) of sputum microbiota in the severe and critically ill groups. **(B)** Beta diversity (Principal coordinates analysis, PCoA) of sputum microbiota in the severe and critically ill groups. No significant difference of bacterial communities between two groups.

A total of 10,083,115 filtered high-quality partial reads were generated, averaging 157,548 reads per sample. Rarefaction curves depicting sequence numbers per sample demonstrated that the mean number of sequences attained a plateau around ~5000 sequence reads (**Supplementary Figure 1**). This observation indicated comprehensive taxonomy detection within each group, and that 5000 reads sufficed to identify the majority of bacterial community members within the sputum microbiota. Evaluation of alpha diversity (Simpson and Shannon indices) and richness (Chao1, ACE) indicated comparable levels of diversity in the sputum microbiota of both groups (**Figure 1A**). PCoA of Bray-Curtis matrices revealed no significant differentiation between the two groups, underscoring the similarity in beta diversity (PERMANOVA, pseudo-F: 0.917, *p*=0.549, **Figure 1B**).

### 3.3 Bacterial taxonomic differences between severe and critically ill influenza groups

**Figure 2:**
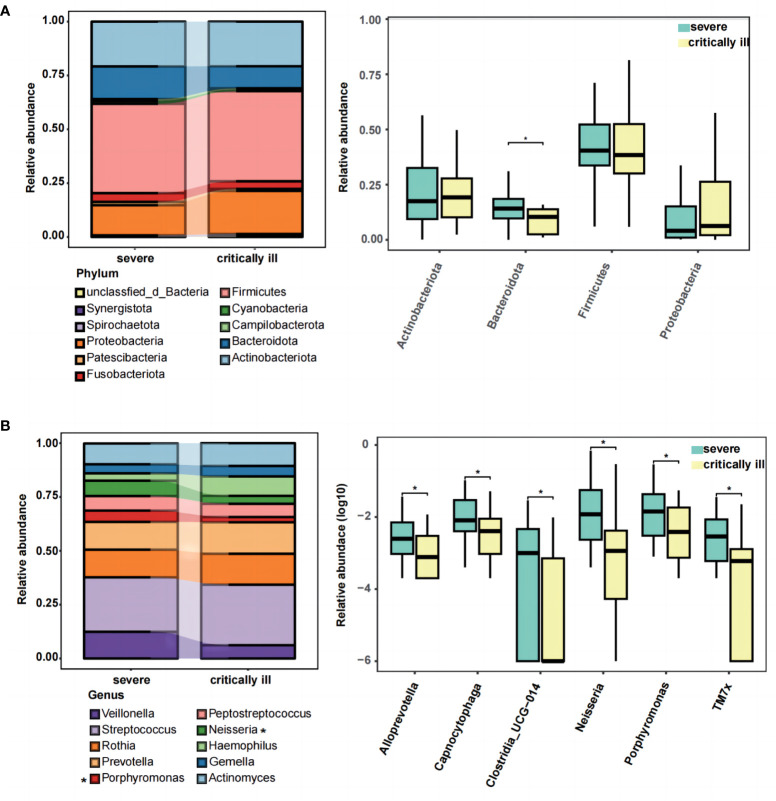
The composition and difference of sputum microbial taxa between the severe and critically ill influenza patients at the phylum and genus levels. **(A)** The top ten phylum of bacteria in relative abundance (left), and the Wilcoxon Rank Sum test was performed to detect taxa with significant differences in relative abundances at the phylum levels between the two groups (right). **(B)** The top ten genus of bacteria in relative abundance (left), and the Wilcoxon Rank Sum test was performed to detect taxa with significant differences in relative abundances at the genus levels between the two groups (right). Significant differences are indicated by *p<0.05.

Upon reaching sequence saturation, relative abundance conversion was executed, retaining phylum and genus-level taxonomies with relative abundance surpassing 1% in any given sample. This process yielded 11 qualified phylum-level and 72 genus-level taxonomies. The top five predominant phyla in the severe group were *Firmicutes* (41.5% of total reads), *Actinobacteriota* (20.8%), *Proteobacteria* (14.0%), *Bacteroidota* (15.2%), and *Fusobacteriota* (4.1%) (**Figure 2A**, left). The critically ill group exhibited *Firmicutes* (41.8% of total reads), *Actinobacteriota* (20.7%), *Bacteroidota* (10.2%), *Proteobacteria* (20.1%), and *Fusobacteriota* (3.6%) (**Figure 2A**, left). The remaining phyla constituted relatively lower abundances (less than 1.5%). A phylum-level analysis revealed a statistically significant decrease in *Bacteroidota* (mean_critically ill_=0.102, mean_severe_=0.152, *p*=0.031) within the critically ill group, whereas *Proteobacteria* (mean_critically ill_=0.201, mean_severe_=0.140) demonstrated an increase, though without statistical significance (**Figure 2A**, right). At the genus level, we presented the distribution of the top 10 relative abundance bacteria in the severe and critically ill groups, and found that *Streptococcus* (mean_critically ill_=0.281, mean_severe_=0.253) and *Haemophilus* (mean_critically ill_=0.090, mean_severe_=0.034) displayed augmentation within the critically ill group, while *Neisseria* (mean_critically ill_=0.037, mean_severe_=0.071), *Veillonella* (mean_critically ill_=0.062, mean_severe_=0.124), and *Porphyromonas* (mean_critical illness_=0.024, mean_severe_=0.053) exhibited reduction (**Figure 2B** left, **Supplementary Figure 3B**). Further to screen for differential bacteria, a comprehensive Wilcoxon test was performed on all genera, elucidating significant differences in *Neisseria* and *Porphyromonas* (*p*=0.012 and 0.037) (**Figure 2B**, right). Furthermore, within the critically ill group, a decrease was observed in *Alloprevotella*, *Capnocytophaga*, *Clostridia_UCG-014*, and *TM7x* (*p*=0.048, 0.049, 0.043, 0.020, respectively) (**Figure 2B**, right).

**Figure 3:**
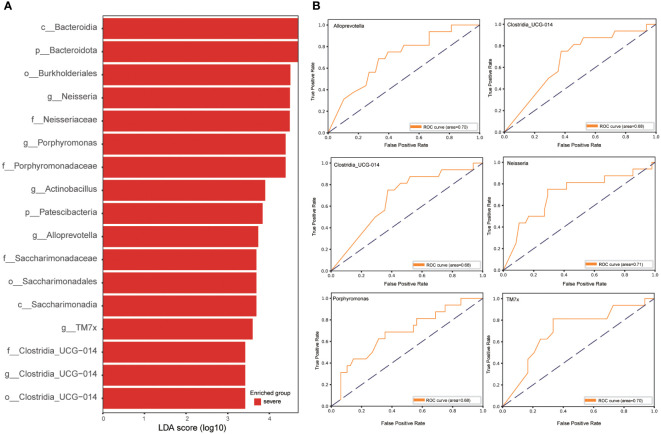
Linear discriminative analysis effect size (LEfSe) analysis in the severe and critically ill influenza patients. **(A)** LDA scores indicate significant differences in the microbiota between the severe and critically ill influenza patients. **(B)** Prediction of the key genera in the severe and critically ill influenza patients. Receiver-operating characteristic (ROC) plot for *Neisseria*, *Porphyromonas*, *Actinobacillus*, *Alloprevotella*, *TM7x*, *Clostridia_UCG-014*, area under the parametric curve (AUC) value=0.71, 0.68, 0.60, 0.70, 0.70 and 0.68 respectively.

To ascertain pivotal phylotypes contributing to the differentiation of sputum microbiota between the two groups, LEfSe analysis was executed, setting a threshold of 2. The sputum microbiomes of the severe group were characterized by an abundance of *Neisseria*, *Porphyromonas*, *Actinobacillus*, *Alloprevotella*, *TM7x*, and *Clostridia_UCG-014*, exhibiting ROC-plot AUC values of 0.71, 0.68, 0.60, 0.70, 0.70, and 0.68 respectively (**Figures 3A, B**).

### 3.4 Correlation between sputum microbiota and influenza Ct values

**Figure 4:**
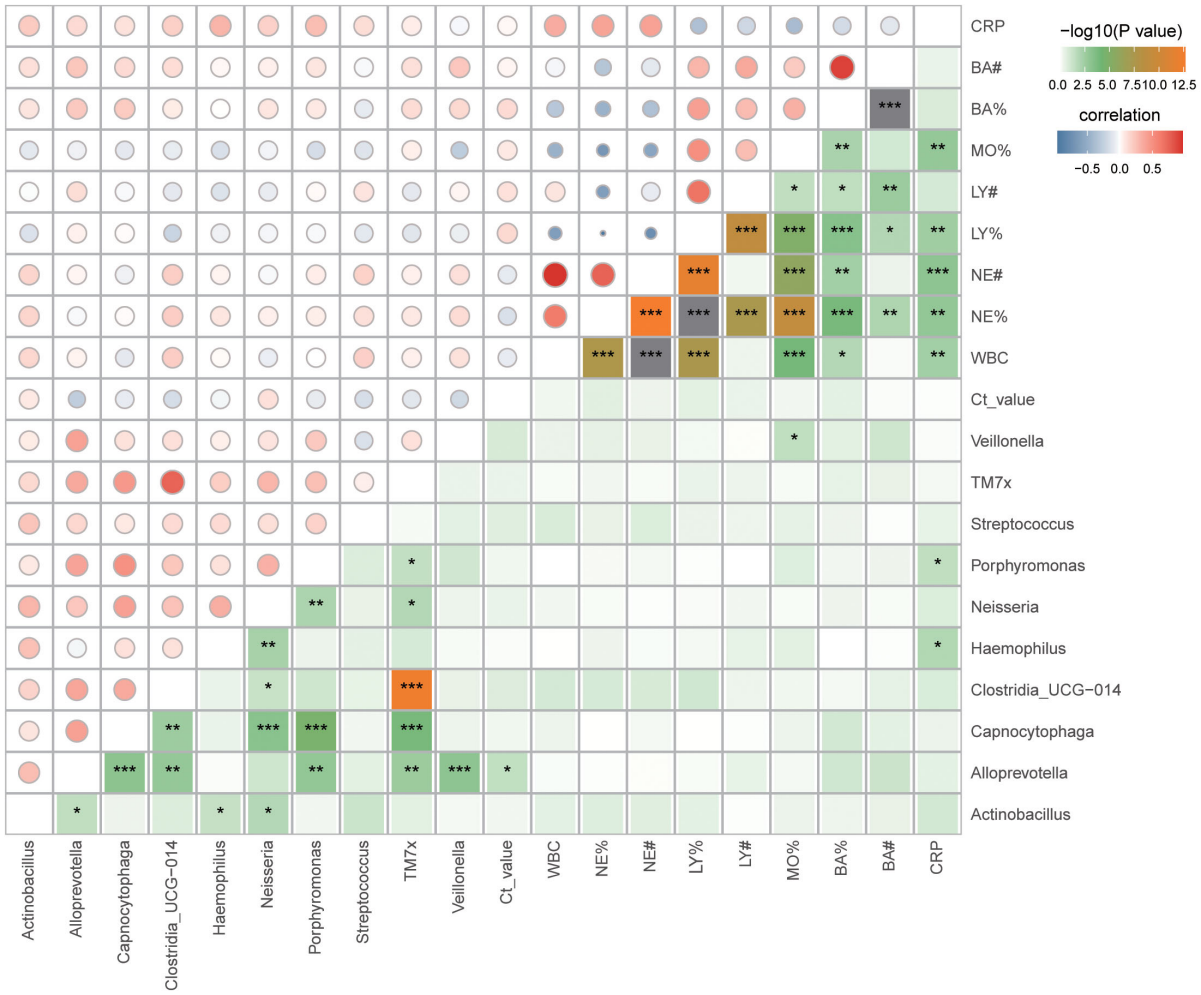
Correlation between sputum microbiota and clinical data. Spearman correlation analysis, *p < 0.05; **p < 0.01; ***p< 0.001. WBC, White Blood Cell; NE%, Neutrophil percentage; NE#, Neutrophil count; LY%, Lymphocyte percentage; LY#, Lymphocyte count; MO%, Monocytes percentage; BA%, Basophil percentage; BA#, Basophil count; CRP, C-reactive protein.

16S rRNA gene analysis of sputum samples indicated a prevalence of *Streptococcus* and *Haemophilus* in the critically ill group, and *Neisseria*, *Porphyromonas*, *Actinobacillus*, *Alloprevotella*, *TM7x*, *Clostridia_UCG-014*, *Capnocytophaga*, and *Veillonella* in the severe group. Spearman correlation analysis was performed to investigate the relationship between these genera, Ct value, and laboratory examinations (WBC, NE%, NE#, LY%, LY#, MO%, BA%, BA#, CRP). The outcomes revealed a negative correlation between *Alloprevotella* and Ct value. Additionally, *Haemophilus* and *Porphyromonas* exhibited positive correlations with CRP (**Figure 4**; **Supplementary Figure 3A**; **Supplementary Table 1**). Meanwhile, *Alloprevotella*, *Capnocytophaga* and *Porphyromonas* were negatively correlated with hospitalization days. (**Supplementary Figure 4**; **Supplementary Table 2**).

## 4 Discussion

The respiratory tract, with its distinct microbial communities in various segments, may respond to IAV infection in both similar or distinct ways. While studies often employ mouse models to depict short- or long-term microecological imbalances post IAV infection (Yildiz et al., 2018), clinical cohorts remain underexplored as starting points to decipher post-influenza respiratory tract microbiota changes. Investigations into bacterial microbiota within the URT of influenza patients, spanning oropharynx, nasopharynx, and other segments, have been conducted (Chen et al., 2023). However, limited inquiry has been dedicated to microbial shifts in the LRT following influenza infection. Hence, by employing 16S rRNA gene sequencing, we undertook a comparative analysis of LRT microbiota (sputum) between severe and critically ill patients. Additionally, we assessed the relationship between microbiota and influenza virus Ct values and laboratory examinations.

No marked disparity in alpha and beta diversity was observed in sputum microbiota between severe and critically ill patients in this study. In animal models, IAV-infected mice showed minimal alterations in microbial diversity and richness within the upper and lower respiratory tracts and even different respiratory segments (Planet et al., 2016; Yildiz et al., 2018; Chen et al., 2023). Clinical research, however, has yielded diverse results. For instance, Shannon diversity was significantly lower in influenza A and B groups in comparison to non-influenza groups (nasopharyngeal swab samples) (Rattanaburi et al., 2022), but higher in H7N9 patients relative to healthy controls (oropharyngeal swab samples) (Lu et al., 2017). Among severe pneumonia patients who were influenza virus positive and negative, no statistical difference was observed in any of the Chao1 or Shannon and Simpson indexes (bronchoalveolar lavage fluid specimens) (Zhou et al., 2023). In our study, sputum samples were used as most severe influenza patients can cough and expectorate, thus making it feasible to collect sputum samples in a non-invasive manner. Although influenza A was identified in the samples, influenza subtypes were not distinguished. Hence, these disparities could be attributed to variant viral strains and different respiratory tract sampling sites and methods.

At the phylum level, researcher has previously observed that the URT microbiota of H1N1 influenza patients were mainly composed of *Actinobacteria*, *Firmicutes*, and *Proteobacteria* (Chaban et al., 2013). Our study found that the same applies to LRT microbiota. Notably, *Bacteroidetes* was dominant in the severe group. During IAV infection’s acute and recovery stages, *Gammaproteobacteria*, *Firmicutes*, and *Bacteroides* class relative abundance escalates within the LRT (Gu et al., 2019). In line with these findings, our results indicated a prevalence of *Streptococcus* and *Haemophilus* in the critically ill group (although there was no significant difference compared with the severe group), corresponding to *Firmicutes* and *Gammaproteobacteria*, respectively. The species of these bacteria are found in the URT of healthy individuals and can potentially cause respiratory infections or influenza virus infections (Ohara-Nemoto et al., 2008; Zhang et al., 2020). Interestingly, significant dissimilarities were evident in specific bacterial taxa, particularly within the severe influenza group. Our research pinpointed variations in *Neisseria*, *Porphyromonas*, *Actinobacillus*, *Alloprevotella*, *TM7x*, *Clostridia_UCG-014*, *Capnocytophaga*, and *Veillonella*. Recently, *Veillonella* has emerged as a noteworthy biomarker for various respiratory viral infections (Li et al., 2023). Dysbiotic microbiota characterized by reduced microbial diversity and elevated abundance of specific bacteria, such as *Streptococcus*, *Pseudomonas*, and *Neisseria*, have been documented in patients with respiratory virus infections (Porto and Moraes, 2021). The link between IAV and *Neisseria* meningitidis disease has been established, and *Neisseria* meningitidis has been found to enhance IAV infection by adhering to human HEC-1-B epithelial cells (Rameix-Welti et al., 2009).

The results of our data showed that there were no statistically significant differences between the two groups in the history of antiviral therapy before the current admission, as well as in the days from the onset of illness to admission, the days from the onset of illness to specimen collection, and the length of hospital stay. However, our Spearman correlation analysis showed that *Alloprevotella* and *Porphyromonas* were negatively correlated with hospitalization days, respectively. In patients with COVID-19, poor clinical outcome was associated with the enrichment of an oral commensal (*Mycoplasma salivarium*) in the lower airways (Sulaiman et al., 2021). These suggest that the characteristics of the microbiota may, to some extent, be indicative of disease progression in patients with respiratory viruses. Our investigation identified elevated WBC, NE%, and NE# in the critically ill group relative to the severe group, coupled with reduced LY% and LY#. Previous research has similarly indicated a higher proportion of neutrophils in critically ill influenza cases compared to non-critically ill cases, with a correspondingly lower lymphocyte proportion (Li et al., 2022). Our findings are in accordance with these observations. Although CRP and Ct values exhibited no statistically significant differences between the groups, the critically ill influenza patients manifested elevated CRP levels and lower Ct values, implying heightened viral loads and more pronounced inflammatory reactions or tissue damage. Remarkably, our Spearman correlation analysis between influenza Ct values and the aforementioned genera exposed a negative correlation with *Alloprevotella*. A prior study revealed associations between nose/throat microbiota and susceptibility to influenza virus infection, particularly in terms of the relative abundances of *Alloprevotella* oligotypes(Lee et al., 2019b). As such, we should pay attention to the changes of *Alloprevotella* in the course of influenza infection and its correlation with the severity of the disease. Our study additionally suggests a potential link between *Alloprevotella* and influenza disease severity. For example, CRP exhibited positive correlations with *Haemophilus* and *Porphyromonas*. In cases of chronic obstructive pulmonary disease exacerbations, higher CRP levels are observed in bacterial infections involving *Haemophilus influenzae* and *Streptococcus pneumoniae* (Gallego et al., 2016). Other investigations have also suggested *Porphyromonas* genus as a potential focus in various pulmonary conditions (Guilloux et al., 2021).

While acknowledging our study’s strengths, certain limitations warrant consideration. First, we did not perform testing for other types of respiratory viruses on the sputum samples used in our study. To compensate for this shortcoming to the best of our ability, we meticulously reviewed all tests for respiratory pathogens other than the influenza virus that were conducted during the hospitalization of the patients, and found that the patients were infected with other Gram-negative bacilli, suggesting that influenza virus infection may weaken the immune system and make individuals more susceptible to other bacterial infections. Second, healthy sputum samples are challenging to collect, precluding inclusion of healthy controls. Our study primarily focused on comparing respiratory tract microbiota differences in influenza patients of varying severities. Additionally, our cross-sectional design and limited clinical sample size emphasize the necessity for expanded samples and longitudinal cohort studies. Certain classical limitations of the cross-sectional study design should be acknowledged, such as it not allowing for conclusions of causality. We also employed amplicon-based 16S rRNA gene sequencing, which provides modest taxonomic resolution at the species level. Enhanced classification precision and reproducible metagenomic sequencing are pivotal to validate our approach across multiple longitudinal cohorts and clinical contexts. From the perspective of microbiota, investigating how the results could guide clinical management strategies, such as targeted interventions and personalized treatment approaches should be considered. Manipulating the composition of respiratory tract microbiota in an attempt to impact disease outcomes or treatment responses is also an area of future research. Ultimately, further validation experiments are needed to determine whether these results could be leveraged to delineate future biomarkers that identify patients at risk of progression to critically ill states.

## 5 Conclusions

The outcomes of this study bestow foundational insights for prospective investigations delving into the intricate interplay between the LRT microbiota and respiratory diseases. Furthermore, additional scrutiny is warranted to unravel the underlying mechanisms governing the interplay between IAV and *Alloprevotella* in disease progression. The innovation encapsulated within this study augments our understanding of the intricate host-microbiota-virus dynamics, accentuating the goal of clinical cohort-driven inquiries into microbiota shifts during viral infections.

## Data availability statement

The data presented in the study are deposited in the National Center of Biotechnology Information (NCBI) repository, accession number PRJNA1045822.

## Ethics statement

The studies involving human participants were reviewed and approved by Beijing Ditan Hospital. The patients/participants provided their written informed consent to participate in this study.

## Author contributions

ZG: Conceptualization, Data curation, Formal Analysis, Investigation, Methodology, Project administration, Resources, Visualization, Writing – original draft, Writing – review & editing. YZ: Conceptualization, Data curation, Formal Analysis, Investigation, Methodology, Project administration, Resources, Supervision, Visualization, Writing – review & editing. XZ: Conceptualization, Data curation, Investigation, Project administration, Resources, Writing – review & editing. TL: Conceptualization, Data curation, Formal Analysis, Investigation, Methodology, Project administration, Resources, Visualization, Writing – review & editing. SS: Conceptualization, Data curation, Investigation, Project administration, Resources, Writing – review & editing. RS: Conceptualization, Data curation, Formal Analysis, Investigation, Methodology, Project administration, Resources, Supervision, Visualization, Writing – review & editing. RJ: Conceptualization, Data curation, Formal Analysis, Investigation, Methodology, Project administration, Resources, Supervision, Visualization, Writing – review & editing.

## Supplementary material

Supplementary Figure 1 Rarefaction analysis of bacterial 16S rRNA gene sequences. Pielou and Shannon indexs were used to evaluate whether further sequencing would likely detect additional taxa, indicated by a plateau.

Supplementary Figure 2 Comparison of clinical indicators between severe and critically illness influenza patients. **(A)** CRP: C-reactive protein; **(B)** LY%: Lymphocyte percentage; **(C)** LY#: Lymphocyte count; **(D)** MO%: Monocytes percentage; **(E)** NE%: Neutrophil percentage; **(F)** NE#: Neutrophil count; **(G)** WBC: White Blood Cell; **(H)** BA#: Basophil count; **(I)** BA%: Basophil percentage.

Supplementary Figure 3 Distribution of *Haemophilus* in severe and critically ill influenza patients **(A)** and its correlation with C-reactive protein (CRP) **(B)**.

Supplementary Figure 4 Correlation between sputum microbiota and clinically relevant days.
