# Supplementary figures and images for: Comparing sputum microbiota characteristics between severe and critically ill influenza patients
Source: Front Cell Infect Microbiol. 2023 Dec 18;13:1297946. doi: 10.3389/fcimb.2023.1297946 (PMC10766813; doi:10.3389/fcimb.2023.1297946)

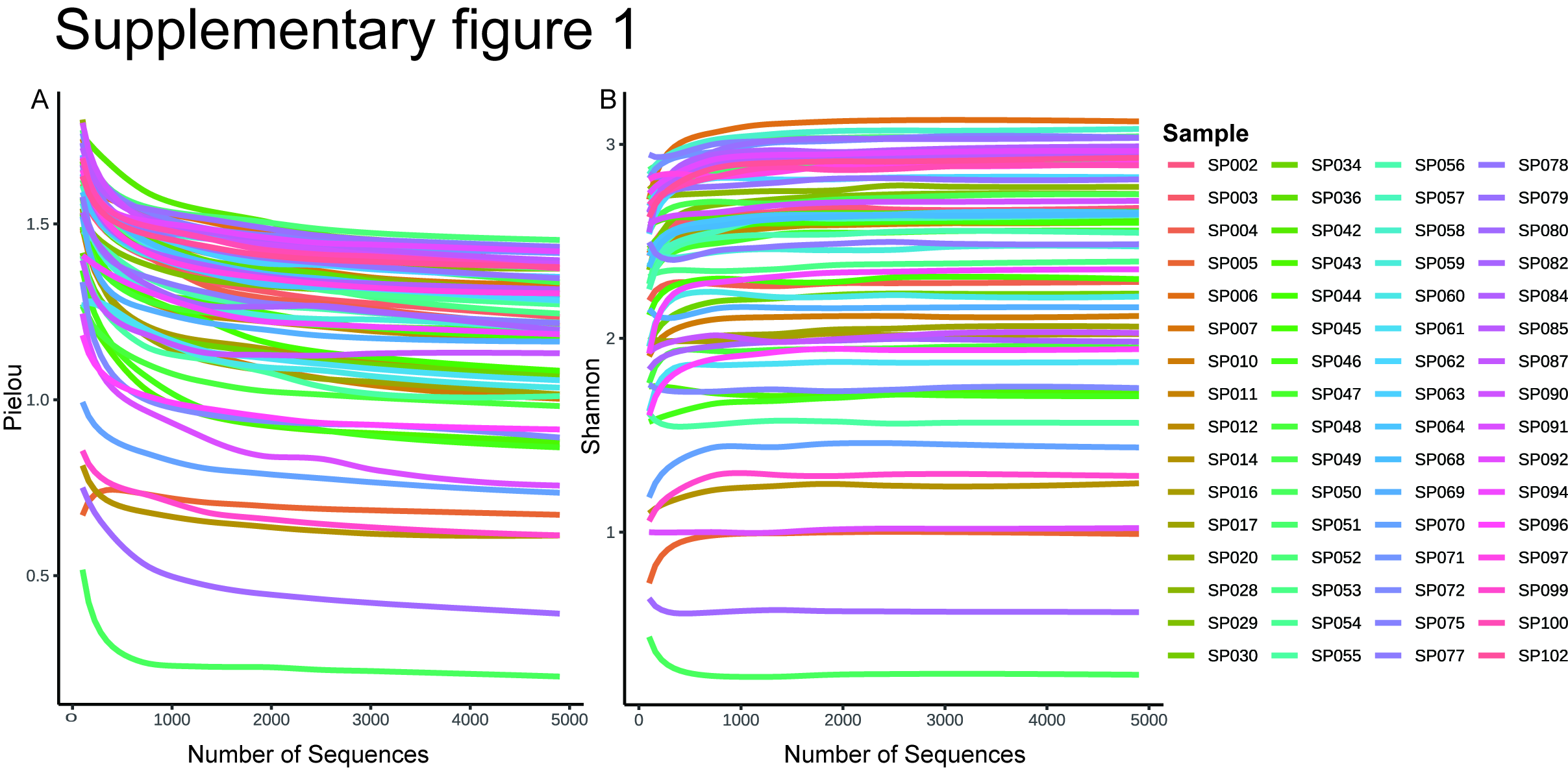

Supplement: Supplementary Figure 1 — Rarefaction analysis of bacterial 16S rRNA gene sequences. Pielou and Shannon indexs were used to evaluate whether further sequencing would likely detect additional taxa, indicated by a plateau. [file Image_1.tif]

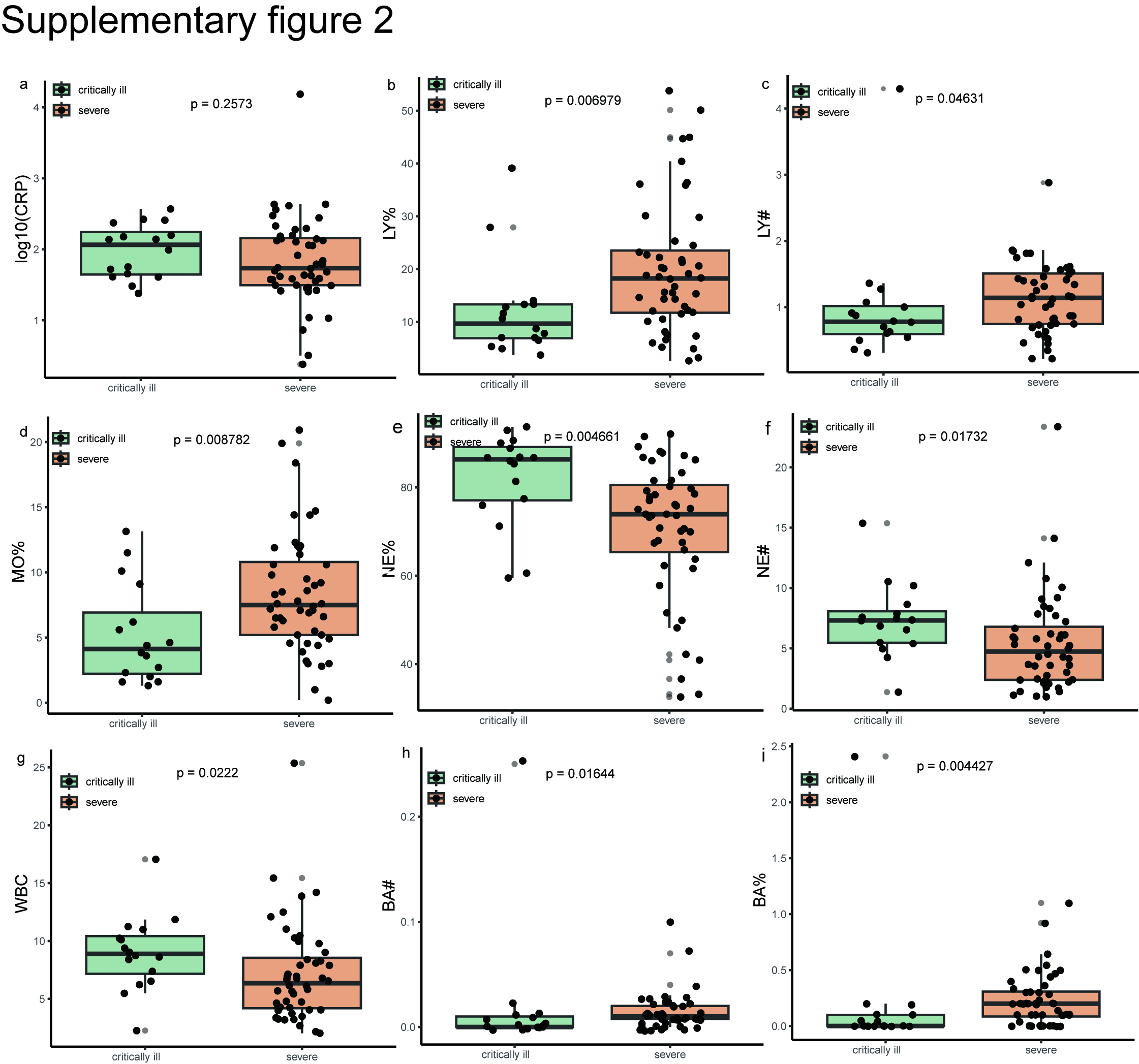

Supplement: Supplementary Figure 2 — Comparison of clinical indicators between severe and critically illness influenza patients. (A) CRP: C-reactive protein; (B) LY%: Lymphocyte percentage; (C) LY#: Lymphocyte count; (D) MO%: Monocytes percentage; (E) NE%: Neutrophil percentage; (F) NE#: Neutrophil count; (G) WBC: White Blood Cell; (H) BA#: Basophil count; (I) BA%: Basophil percentage. [file Image_2.tif]

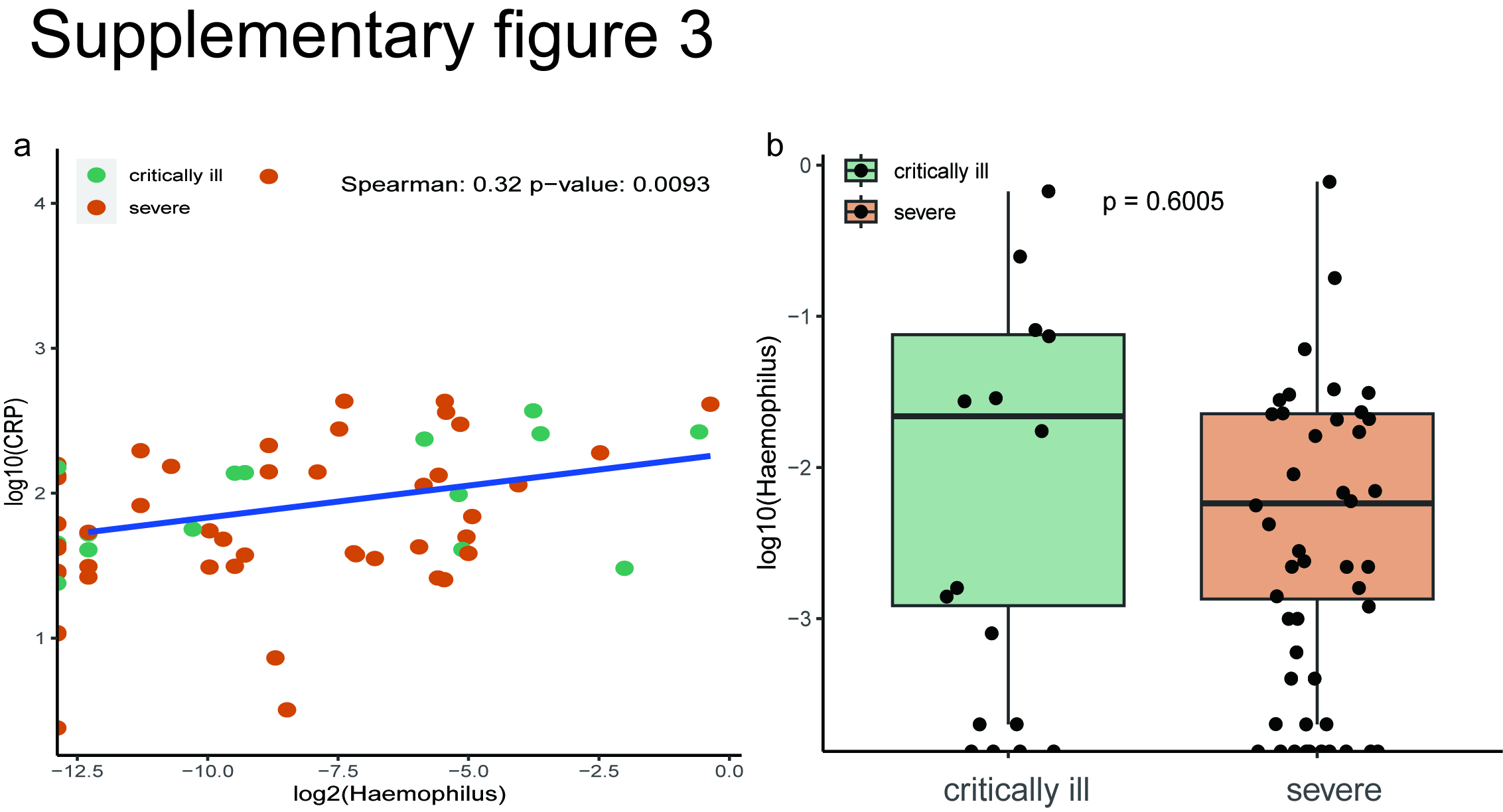

Supplement: Supplementary Figure 3 — Distribution of Haemophilus in severe and critically ill influenza patients (A) and its correlation with C-reactive protein (CRP) (B). [file Image_3.tif]

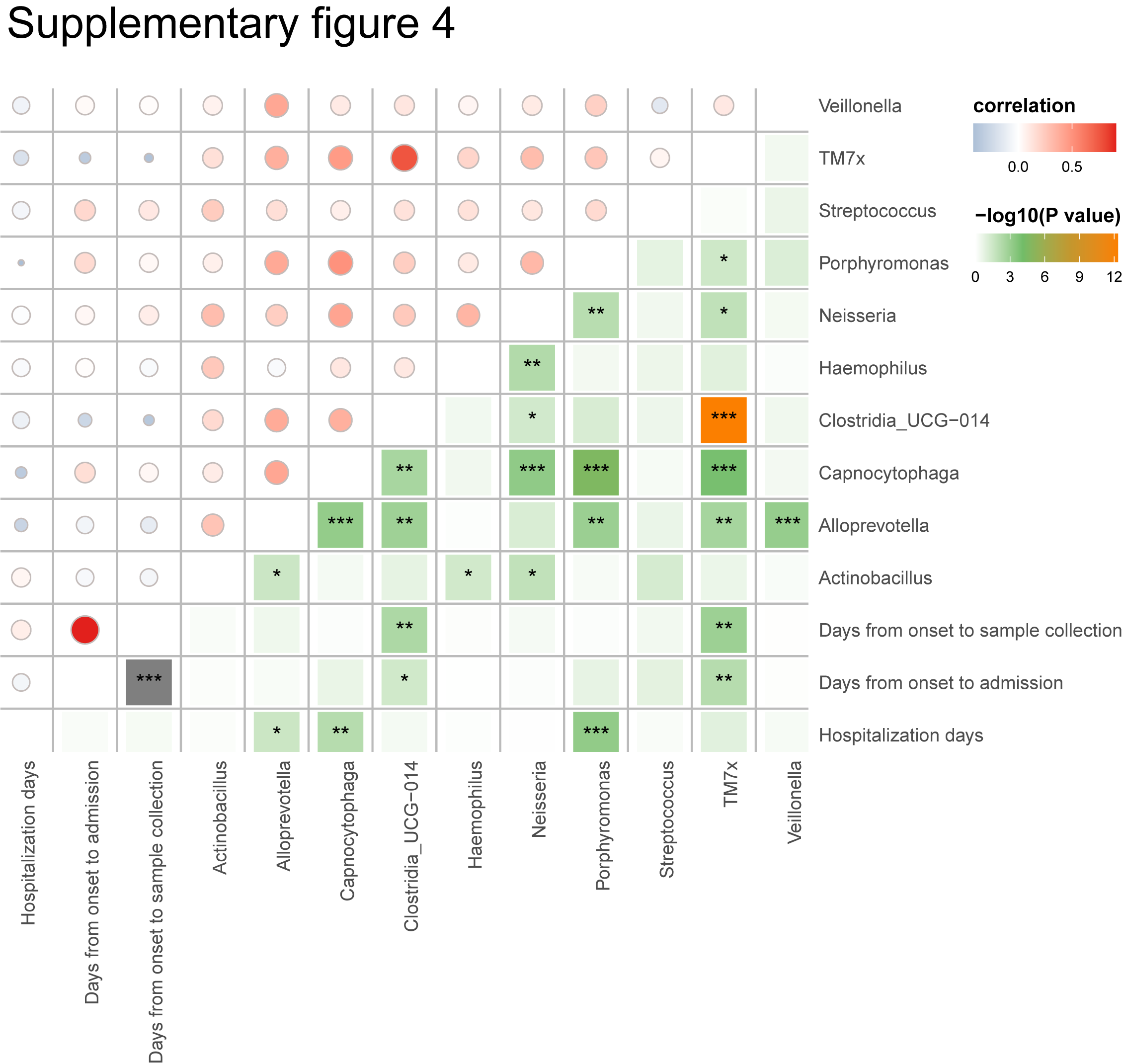

Supplement: Supplementary Figure 4 — Correlation between sputum microbiota and clinically relevant days. [file Image_4.tif]
